# The future of brachytherapy physics: Innovation, quality, and the next generation of clinical practice

**DOI:** 10.1002/acm2.70834

**Published:** 2026-09-28

**Authors:** Nataliya Kovalchuk, Åsa Carlsson Tedgren, Frank‐André Siebert, Mark J. Rivard, Alexandra Rink, Yi Rong

**Affiliations:** ^1^ Radiation Oncology Department Stanford University Stanford California USA; ^2^ Department of Oncology Pathology Karolinska Institutet Stockholm Sweden; ^3^ Department of Nuclear Medicine and Medical Physics Karolinska University Hospital Stockholm Sweden; ^4^ Department of Health, Medicine and Caring Sciences Linköping University Linköping Sweden; ^5^ Medical Physics Department UKSH, Campus Kiel Kiel Germany; ^6^ Department of Radiation Oncology Brown University Providence Rhode Island USA; ^7^ Radiation Medicine Program Princess Margaret Caner Centre, University Health Network Toronto Ontario Canada; ^8^ Department of Radiation Oncology University of Toronto Toronto Ontario Canada; ^9^ Department of Medical Biophysics University of Toronto Toronto Ontario Canada; ^10^ Radiation Oncology Department Mayo Clinic Phoenix Arizona USA

Soon after the discovery of radionuclides, brachytherapy was first applied with ^226^Ra to treat skin cancer in 1903, and has evolved rapidly over the past 120 years.^1^ Brachytherapy remains one of the most conformal and biologically effective forms of radiation therapy, providing sharp dose fall‐off and minimizing dose to surrounding normal tissues.^2^, ^3^, ^4^ Despite decades of clinical evidence supporting its efficacy especially for cervical cancer patients, brachytherapy faces important challenges with declining utilization in some regions, workforce shortages, increasing procedural complexity, and unequal global access.^5^, ^6^, ^7^, ^8^ A recent global demand and supply study published in The Lancet Oncology revealed that brachytherapy demand was met in most high‐income countries (81.5%), but only 44.4% for upper‐middle‐income countries and 11.8% for lower‐middle‐income countries.^7^ Global access to brachytherapy remains highly unequal, and continued technological development happening in those high‐income countries may widen this disparity. Meanwhile, rapid advances in imaging, model‐based dose calculation algorithms (MDBCAs),^9^ 3D‐printing, artificial intelligence (AI), automation, and electro‐magnetic tracking are reshaping how brachytherapy is planned and delivered.^10^, ^11^, ^12^, ^13^, ^14^

The future of brachytherapy will depend not only on technological innovation but also on maintaining rigorous physics standards, educating the next generation of medical physicists, and ensuring that advances translate into safe, efficient, and equitable patient care worldwide.

In recognition of the World Brachytherapy Awareness Day (July 17^th^), we dedicated this Global Spotlight editorial to four internationally recognized leaders in brachytherapy physics: Dr. Åsa Carlsson Tedgren (Sweden), Dr. Frank‐André Siebert (Germany), Dr. Alexandra Rink (Canada), and Dr. Mark Rivard (United States) to share their perspectives on the future of brachytherapy, emerging technologies, and the increasing need for brachytherapy physics expertise globally.

**Dr. Åsa Carlsson Tedgren** (Figure 1) is an Adjunct Professor of Medical Radiation Physics at Karolinska Institutet, a Medical Physicist at Karolinska University Hospital, and affiliated to Linköping University in Sweden. She is internationally recognized for her work in brachytherapy dosimetry, Monte Carlo modeling, treatment planning, and quality assurance. Her research has advanced the understanding of dose calculation accuracy in brachytherapy and has contributed to improving clinical implementation of modern treatment planning techniques. She has authored numerous peer‐reviewed publications in leading medical physics and radiation oncology journals, including the Journal of Applied Clinical Medical Physics. Beyond her scientific contributions, Dr. Carlsson Tedgren has played a major role in medical physics education and international collaboration through ESTRO, AAPM, and other professional organizations.

1. Dr. Tedgren, brachytherapy has seen remarkable technological advances over the past two decades, including 3D image‐guided adaptive treatments, MBDCAs, and increasingly sophisticated treatment planning systems (TPSs). Which innovation do you believe has had the greatest impact on clinical practice, and what emerging technology do you think will define the next decade?
  I believe that 3D image guidance has had the greatest impact on brachytherapy clinical practice over the past two decades. It has enabled treatment planning based on individual patient anatomy and improved our ability to define targets and organs‐at‐risk (OARs) and to evaluate the resulting dose distributions. In gynecological brachytherapy, repeated imaging and treatment planning have also enabled an adaptive approach, accounting for anatomical changes during the course of treatment.

Looking ahead, I believe increased automation, increasingly supported by AI, has the potential to be one of the most transformative developments in brachytherapy, by reducing many manual and time‐consuming steps in current workflows and making some processes less dependent on individual operator experience. I also see treatment verification as an important area for development. Brachytherapy remains a highly procedure‐dependent modality in which discrepancies between the planned and actually delivered treatment may go undetected. Improved verification could therefore provide an important additional layer of safety.

2. AI and automation are beginning to influence contouring, applicator reconstruction, treatment planning, and quality assurance. Which aspects of brachytherapy do you believe are most likely to benefit from AI, and where do you think expert human judgment will remain indispensable?
  I see considerable potential for increased automation within brachytherapy treatment planning, with AI providing new tools alongside continued developments in optimization methods and physics‐based models. Contouring, applicator and catheter reconstruction, and treatment plan optimization all involve substantial manual work today; increased automation can improve efficiency and reduce unwanted operator dependence. AI may also contribute more broadly to diagnosis, treatment selection, and follow‐up through the integration and analysis of imaging and other patient data. At the same time, I believe it is essential that automation does not replace understanding. Medical physicists using automated tools must understand the underlying principles, be able to judge whether the results are reasonable, recognize situations in which an algorithm may fail, and know how to intervene. Maintaining this expertise may itself become a challenge as automation increases, since skills and understanding are difficult to preserve when the underlying tasks are rarely performed manually.
3. Many countries face shortages of experienced brachytherapy physicists while the complexity of treatments continues to increase. How should education and training evolve to prepare the next generation of medical physicists for the future of brachytherapy?
  Education and training are essential in brachytherapy. As technology and workflows evolve, medical physicists need both a strong understanding of the underlying physics and practical experience of the complete clinical workflow. Well‐defined training objectives and curricula are important to ensure that core brachytherapy knowledge and skills are maintained as new technologies and increasing automation are introduced. Formal courses and online education have an important role, but I do not think they can fully replace hands‐on training. Training at an experienced brachytherapy center provides exposure to procedures, practical problem‐solving, and clinical situations that are difficult to learn from guidelines or lectures alone. Exchange between clinics, including shorter visits, can also be valuable by providing insight into different workflows and approaches. Structured opportunities for such practical training and exchange should therefore be supported for medical physicists. A major challenge is that workforce shortages also make education and training more difficult. Releasing staff for courses, exchanges, or training visits requires time and resources, and course and travel costs can also be substantial. Developing complementary and accessible forms of education, while maintaining opportunities for practical training and exchange between centers, will therefore be important for the next generation of brachytherapy physicists.
4. As we learned from the recent Lancet publication on the global demand and supply in brachytherapy resources and access,^7^ many patients in the low‐ and middle‐income countries struggle to have access to receive brachytherapy treatment. From your perspective, what practical steps should the global brachytherapy community take to ensure that technology advancements narrow rather than widen such disparities?
  The profound inequalities in brachytherapy access demonstrated by Hajeer et al. call for investment not only in new centers, but also in the expertise required to establish and sustain safe, high‐quality brachytherapy services. International collaboration can contribute through education, exchange between centers, and practical assistance when new programs are established. Initiatives such as BrachyTerra^15^ provide an excellent example of how high‐quality education can be made freely accessible and adapted to regional requirements. I also believe that technological goals need to be realistic and adapted to local needs and available resources. The aim should not necessarily be to reproduce every technological advance implemented in the most resource‐rich centers, but to identify developments that provide the greatest clinical benefit while maintaining safety and quality. For example, although MRI provides important advantages for image‐guided cervical brachytherapy, CT‐based treatment planning can enable high‐quality 3D image‐guided brachytherapy where access to MRI is limited. Further software automation may also help make modern brachytherapy workflows more feasible in settings with limited staffing, by reducing some of the time‐consuming manual steps in treatment planning.

**FIGURE 1:**
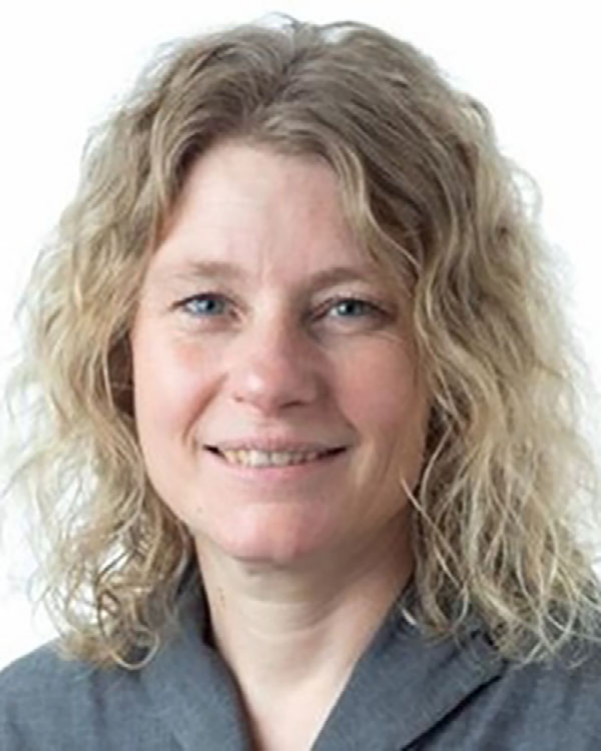
Åsa Carlsson Tedgren.

**Dr. Frank‐André Siebert** (Figure 2) is a Professor of Medical Physics and the Head of Medical Physics Department UKSH, Campus Kiel, Germany and an internationally recognized expert in brachytherapy dosimetry and treatment planning. His work has significantly contributed to the development and clinical implementation of model‐based dose calculation algorithms, Monte Carlo methods, and modern brachytherapy physics standards. He has authored numerous peer‐reviewed publications in leading medical physics and radiation oncology journals, including the Journal of Applied Clinical Medical Physics. He has also served on numerous national and international committees (e.g. chair of the GEC‐ESTRO committee from 2024 to 2026) and has helped shape recommendations that influence clinical brachytherapy practice worldwide.

1. Dr. Siebert, over the past two decades, GEC‐ESTRO has produced landmark recommendations that have standardized many aspects of brachytherapy physics worldwide. From your perspective, what has been the greatest achievement of these collaborative efforts, and where do you think international consensus is still needed?
  GEC‐ESTRO has a long tradition of developing guidelines on clinical and physics‐related topics in brachytherapy. One important achievement is that different working groups within GEC‐ESTRO collaborate on guidelines, thereby combining their expertise in particular for clinical recommendations. For example, the brachytherapy guidelines for prostate cancer were developed by members of UroGEC and the physics working group BRAPHYQS. Currently, guidelines are also being prepared in collaboration with BRAPHYQS and the Head & Neck & Skin working group. In addition, we have several recommendations focusing specifically on physics‐related aspects. The dosimetry guidelines for low‐dose rate (LDR) and high‐dose rate (HDR) photon‐emitting sources, for example, are highly relevant in clinical practice. I would also like to highlight the fruitful collaboration with the AAPM. We have published several joint guideline projects with our American colleagues, addressing topics such as uncertainties in brachytherapy, innovative brachytherapy devices, surface brachytherapy, and MBDCAs. Looking ahead, I think it will be important to present guidelines in a well‐organized manner and across societies. This would make it much easier for users to obtain a comprehensive overview of the available recommendations.

You asked what is still needed. In my opinion, there is a particular need for more practical recommendations. For example, guidance on the use of in vivo dosimetry in brachytherapy, the application of biological treatment planning, or image registration would be valuable. Other relevant topics include 3D printing and the use of in‐house software from a regulatory and legal perspective as in Europe, the Medical Device Regulation makes the clinical use of self‐developed products more challenging.

2. Brachytherapy continues to evolve with new imaging technologies, adaptive workflows, and increasingly personalized treatment approaches. How has the role of the medical physicist changed over your career, and what do you expect it to look like ten years from now?
  As technology and brachytherapy techniques have become increasingly complex, the role of the medical physicist has evolved from “only” calculating the treatment plan to taking on a much broader and more overarching role. The medical physicist is often the person who has the best overview of the entire procedure from the technical perspective. This includes applicator placement in the operating room, all aspects of imaging, applicator reconstruction, and in some cases, contouring, as well as treatment planning, plan evaluation, and of course, quality assurance.

At the same time, the individual responsibilities of all staff members involved must be respected. Overall, I think that medical physicists in brachytherapy have a much more important role today than they did in the past. This position should be used to further develop and optimize brachytherapy procedures.

3. Looking ahead, what do you think are the most important steps the radiation therapy community should take to ensure that brachytherapy continues to thrive as a core component of modern radiation oncology?
  Brachytherapy is an integral component of modern radiotherapy, offering unique dosimetric and clinical advantages. Its highly‐localized dose distribution enables high doses to the target while limiting exposure of surrounding healthy tissues and may facilitate organ preservation in selected patients. It should therefore be regarded as an established component of the radiotherapy spectrum instead of a niche technique. One important point of modern radiation oncology is to identify the optimal treatment for each individual patient. Brachytherapy, stereotactic body radiotherapy (SBRT), particle therapy, and other modalities should be considered complementary options rather than universally competing techniques. To realize its full potential, brachytherapy needs greater visibility and should be considered from the outset in the multidisciplinary management of patients with solid tumors. Education and specialized training, centers of excellence, high‐quality clinical trials, and international consensus on treatment techniques and quality assurance are essential. Standardization and automation may further optimize workflows and address limited availability of specialized personnel.

Economic considerations may also become increasingly important. As healthcare systems face growing financial pressure, the resource efficiency and comparatively low infrastructure requirements of brachytherapy may represent a significant advantage. Future developments include the use of brachytherapy for selected reirradiation strategies and its combination with systemic treatments such as immunotherapy. Overall, brachytherapy should be integrated into an individualized treatment framework in which the most appropriate modality is selected for each patient. Its future role will depend on evidence, expertise, visibility, efficiency, and continued technological and clinical innovation.

4. The recent Lancet publication highlighted major global disparities in access to brachytherapy, particularly in low‐ and middle‐income countries.^7^ Which technological developments do you believe have the greatest potential to improve access and reduce these global disparities?
  Technological developments have the potential to reduce disparities in access to and delivery of brachytherapy. However, technological innovation alone is insufficient. Equally important are adequately trained clinical staff and access to a sufficient number of afterloading systems, including in low‐ and middle‐income countries. As demonstrated by Hajeer et al.,^7^ there is a need for such systems in these settings. Reliable access to the radioactive sources required for afterloading devices must also be ensured, as must appropriate imaging facilities, including CT, MRI, and ultrasound which are essential for modern image‐guided brachytherapy.

Emerging technologies may further improve the efficiency and accessibility of brachytherapy. Automation tools, also AI‐based, could reduce the workload for clinical teams and facilitate the implementation of standardized workflows. In parallel, hypofractionated treatment approaches may reduce the number of treatment sessions, thereby decreasing the burden on patients and reducing the need for travel. In addition, I believe that improved coordination of patient pathways through digitally connected tools, combined with online training opportunities for staff, for example using modern virtual reality techniques, could support the acceptance and wider adoption of brachytherapy in low‐ and middle‐income countries. Together, these measures could contribute to making high‐quality brachytherapy more accessible while maintaining appropriate standards of treatment and safety.

**FIGURE 2:**
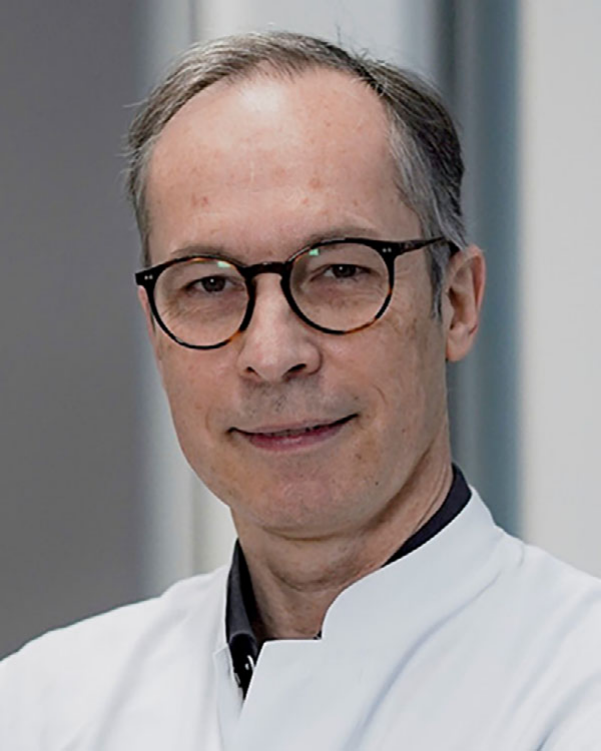
Frank‐André Siebert.

**Dr. Alexandra Rink** (Figure 3) is a Medical Physicist at the Princess Margaret Cancer Centre, an Associate Professor in the Departments of Radiation Oncology and Medical Biophysics at the University of Toronto, and a Clinician Scientist at the Princess Margaret Research Institute. She is an internationally recognized expert in image‐guided brachytherapy, in vivo dosimetry, quality assurance, and interventional radiotherapy. Her work has significantly contributed to the development of optical fiber–based in vivo dosimetry and quality improvement strategies for brachytherapy. She has authored numerous peer‐reviewed publications in leading medical physics and radiation oncology journals, including the Journal of Applied Clinical Medical Physics, where she serves as an Associate Editor. She has also served on numerous national and international committees and has helped advance brachytherapy physics through clinical innovation, research, and education.

1. Dr. Rink, Dr. Carlsson Tedgren highlighted treatment verification as one of the most important future directions in brachytherapy, noting that discrepancies between the planned and delivered treatment may go undetected. Much of your research has focused on real‐time in vivo dosimetry and treatment verification,^16^, ^17^ with a recent venture into more fundamental material science.^18^ What do you see as the major barriers to broader clinical implementation, and how close are we to making real‐time treatment verification a standard component of brachytherapy?
  We invest considerable effort in all the up‐front tasks yet have limited direct‐confirmation that the dose delivered to the patient matches what we intended. Dosimetry should not simply be a quality assurance exercise performed before or after treatment but should ideally become an integral part of the treatment itself. Despite my investigation of new materials, it is hard to imagine that a new material on its own would solve the challenge of in vivo dosimetry in brachytherapy as many sensing technologies already exist. In general, the field is closer technologically than operationally, with major barriers to broader clinical implementation being practicality and interpretability.

First, verification of dose distributions in brachytherapy are challenging given the high dose gradients and small source movements. Further, while in theory dosimeters can be integrated into the applicators to validate treatment delivery,^19^ in vivo dosimetry would still be uncertain due to changing anatomy caused by organ motion and in some instances implant‐induced oedema. The dosimetry system must be sufficiently sensitive to detect clinically meaningful deviations while avoiding false alarms that could undermine confidence in the in vivo technology.^20^, ^21^ This may require guidance or consensus on what and where we should measure, what level of agreement constitutes acceptable delivery, and how detected discrepancies should be handled, especially in procedures where patients remain anesthetized during treatment delivery. Secondly, integration into clinical workflow remains a significant challenge. Dosimetry systems need to provide actionable information without increasing procedure time or complexity. Even a technically excellent dosimeter will struggle to gain adoption if it requires substantial additional effort or specialized expertise to operate and interpret. Demonstrating clinical benefit with systems that are simple, reliable, and validated through multicenter studies will be essential.

2. You have led the implementation of MRI‐guided interstitial brachytherapy and studied the effects of organ motion, workflow efficiency, and treatment uncertainties. Which aspects of image‐guided brachytherapy can be further developed to provide greatest opportunities for improving treatment quality and patient outcomes over the next decade?
  Given the high dose per fraction typically delivered, I believe that treating each image‐guided brachytherapy fraction as an individual adaptive plan, and for gynecological malignancies using interstitial needles to achieve high conformality to the target volume while sparing OARs, is the best combined approach to realize the highest‐quality treatments. Recent evidence from the EMBRACE‐II study demonstrated disease‐specific survival and progression‐free survival of 85% and 73% at 5 years with 5‐year local control and pelvic nodal control of 92% and 94%, respectively.^22^ But this approach is resource intensive, and despite that MR‐guided adaptive HDR brachytherapy for cervical cancer is what I call “the original adaptive radiotherapy”, we have been leapfrogged by EBRT adaptive workflows. The reason is the imaging, planning, or adapting and delivery parts of the workflows are all integrated and automation is built in. Vendor solutions that take this integrated approach exist, but they are nominally built for ultrasound‐guided prostate HDR treatments, leaving the solution to gynecological HDR brachytherapy as a combination of disparate steps and vendors with little optimization or automation help. Integration, especially with MRI to provide superior soft tissue contrast, could further improve outcomes by providing this standard‐of‐care to a larger population.
3. AI is beginning to influence contouring, catheter reconstruction, treatment planning, and workflow optimization. How far do you think we are from a “zero‐click” brachytherapy workflow where most planning steps are automated and the medical physicist role is primarily to review and validate the treatment plan?
  I am not entirely sure we want a “zero‐click” workflow. I am a strong proponent of the human‐in‐the‐loop approach, where people participate in decision making throughout the process, not just reviewing a plan at the end. The plan could be based on erroneous needle reconstruction or contours, which could be both harder to troubleshoot but may also be missed entirely once we become comfortable and instilled with a perhaps false‐sense of security where all the numbers “look good”. That said, similar to the above response for something close to a “zero‐click” workflow, all of the models would have to be integrated into a single workflow, and that's currently not supported through any vendor, leaving individual teams building their own separate processes.
4. Wang et al.^8^ highlighted major global disparities in brachytherapy access and emphasized that expanding capacity requires more than simply purchasing new equipment. Based on your experience implementing advanced image‐guided brachytherapy, what do you believe are the greatest barriers to translate innovation into routine clinical practice worldwide, and how can the global brachytherapy community help overcome them?
  Equipment is a huge part of it. I have seen someone who trained to deliver MR‐guided interstitial gynecological HDR brachytherapy at our institution become disheartened because the cost of the new interstitial applicators back home in Ghana would be prohibitive. Their physicist was also not familiar with the treatment planning approach for combined intracavitary/interstitial implants, and sending over our protocols, presets, and so on is futile given vendor differences. The thing we do poorly is train the whole team to facilitate these translations worldwide. Traditionally, through fellowships we train radiation oncologists, yet brachytherapy is a team sport. It relies on other disciplines to support the procedures in the way that is different from EBRT. Even though there have been more recent initiatives that aim to train a radiation oncology and physics duo, we rarely extend this training to other disciplines that will be supporting brachytherapy, such as therapists, imaging technologists, nursing staff, and so on. Not only do we have no infrastructure to train the entire team all at once, but the institutions typically cannot have their entire team leave work to attend training, especially for any extended period. This is something the global community can work on to overcome.

**FIGURE 3:**
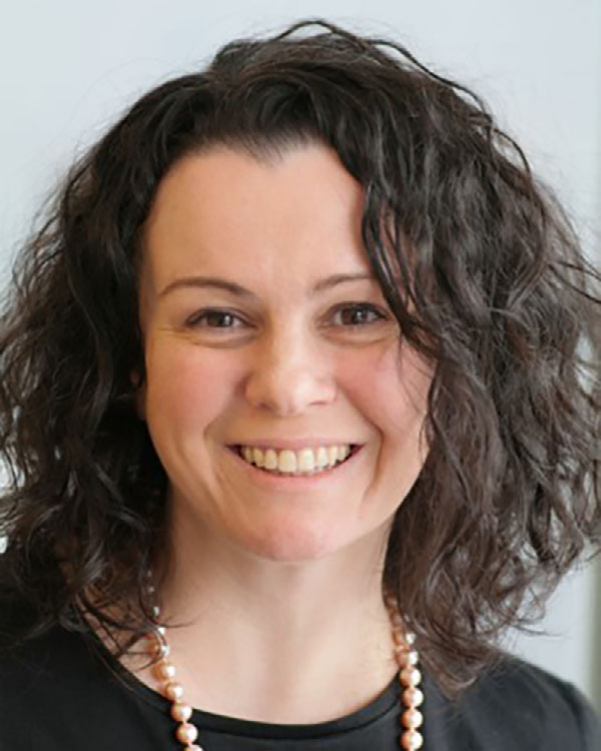
Alexandra Rink.

**Dr. Mark Rivard** (Figure 4) is a Professor of Radiation Oncology at Brown University and the Chief of Brachytherapy Physics at Brown University Health. He is internationally recognized for his contributions to brachytherapy source dosimetry, quality assurance, and clinical implementation. Dr. Rivard has chaired and contributed to numerous AAPM Task Groups that have established widely‐ adopted standards for brachytherapy dosimetry and quality management. He has authored numerous peer‐reviewed publications in leading medical physics and radiation oncology journals, including the Journal of Applied Clinical Medical Physics. His work has had a lasting impact on the safe and effective delivery of brachytherapy worldwide.

1. Dr. Rivard, the TG‐43 and TG‐43 updates have been the cornerstone of clinical brachytherapy dosimetry for decades. Although the TG‐186 Report introduced MBDCAs more than a decade ago, most clinics continue to rely on the TG‐43 formalism for routine treatment planning. What do you see as the major barriers to broader clinical adoption of MBDCAs, and do you believe they will eventually become the new standard of care?
  We can remember how use of material heterogeneity corrections rolled out for EBRT – the advancement was not formally coordinated by professional societies, and manufacturers provided various algorithms at no extra cost. Decades ago, several pioneering institutions developed their own EBRT algorithms and there was competition to offer improved algorithms for the obvious benefit of dosimetric accuracy.

With HDR brachytherapy using high‐energy photon‐emitting radionuclides, the differences between the standard TG‐43 approach and MBDCAs, which account for material heterogeneities and radiation scatter conditions, are usually less than 5% for common treatment sites such as gynecological malignancies, prostate, and skin cancer. Yet brachytherapy TPS vendors currently charge clinics to recoup developmental costs for their MBDCA advancements. The contrasting business models between EBRT and brachytherapy have consequently prevented broad clinical adoption of MBDCAs.

While MBDCA adoption has gradually increased, the field has been in state of flux for several years. Beyond economics, which has become increasingly important globally for radiotherapy, another prominent barrier to MBDCA adoption is computational speed. Dose optimization is still bound to the TG‐43 approach, meaning that a MBDCA calculation is performed after the dwell positions and dwell times have been determined. Ultimately, my hope is for widespread adoption of MBDCAs following modest computational speed improvements to allow optimization, and more generous algorithm availability from the vendors.

2. What do you consider the most important unresolved physics challenges in modern brachytherapy? Are they primarily related to dosimetry, imaging, treatment planning, or something else?
  While the dosimetric characterization of brachytherapy sources is well established, significant opportunities remain to improve dosimetry for low‐energy, LDR brachytherapy. This is true especially under clinical circumstances where there are large differences between the implant geometry and the reference water environment inherent to TG‐43 formalism. Think of prostate calcifications, source proximity to bone, or use of high‐Z collimated shields such as with ocular brachytherapy. Other than the Zeiss Radiance TPS, which is dedicated solely to the INTRABEAM 50 kVp HDR electronic brachytherapy source, there are currently no commercially available TPS using MBDCAs for low‐energy sources such as LDR brachytherapy seeds.

The entire approach to brachytherapy can be reworked to better embrace imaging from a modern perspective. LDR seeds do not visualize under MRI (appearing as empty voids), albeit there are special interseed spacers that clearly show the locations between seeds. Even with detailed societal recommendations, most clinics today still do not incorporate MRI into their brachytherapy clinical workflows. The majority of seeds have dense radiopaque markers designed for compatibility with planar x‐ray imaging from decades ago while causing reconstruction artifacts today under CT that obscure adjacent tissues and blur seed localization accuracy. Yet these uses of imaging are all to guide treatment planning with no widespread verification of actual treatment delivery like portal dosimetry and cone‐beam CT which are available with EBRT. In vivo dosimetry to monitor treatment delivery is the last stage of brachytherapy needed to validate the clinical intent.

Another unresolved issue is the lack of a unified platform for the widespread integration of EBRT and brachytherapy doses, while also accounting for their differences in radiobiology and anatomic positioning. With EBRT we have seen development of plan robustness to bolster treatment delivery, as well as use of deformable image registration to gain a broader view across treatment courses. Brachytherapy would also benefit from these advancements. However, caution is necessary since intrinsically brachytherapy can severely violate tenets of deformable image registration such as conservation of mass due to the presence of applicators, which of course displace tissue and confound even basic dose summation.

We have seen great strides with the use of AI to automate time consuming yet crucial aspects of treatment planning such as contouring. The task of treatment planning is usually performed by medical physicists or dosimetrists, and the TPS manufacturers regularly offer advancements to streamline and automate individual steps. But in recent years, researchers have developed differing approaches to tissue segmentation that surpass simple atlas‐based methods. They have even developed rapid techniques to automate the majority of treatment planning tasks to include synthetic images upon which to perform MBDCA calculations towards immediately jumping to our vision of the future. Of course, much benchmarking and validation of these techniques is necessary, but it seems that in several years the current, lengthy stepwise approach to treatment planning can be behind us. The future is bright from a technological perspective.

3. Brachytherapy offers excellent clinical outcomes for many cancers, yet access remains limited in many clinics because of technical complexity, resource constraints, and workforce challenges. From your perspective, what should be the highest priorities over the next decade to make high‐quality brachytherapy more widely available while preserving the rigorous physics standards that underpin safe and effective treatment?
  Brachytherapy utilization varies markedly across clinics, primarily driven by familiarity and comfort level of the radiation oncologist. A medical physicist cannot unilaterally drive a brachytherapy program; it requires the shared commitment and expertise by the radiation oncologist and the referring physicians. As a form of interventional radiotherapy, brachytherapy can be applied over most all anatomic sites and its use can take many forms in a specific clinic. The easiest sites are for skin with collimated applicators and vaginal irradiation with simple applicators. From there, the complexity of brachytherapy increases with multi‐channel or custom applicators and multi‐needle interstitial implants. The complexity of each brachytherapy technique can take many forms depending on the associated imaging, treatment delivery method, and other related modalities like EBRT or surgery. The medical physicist is responsible for critical technical details such as calibrating the sources of invisible radiation and ensuring spatial and dosimetric accuracy of the treatment planning and the entire treatment delivery.

Currently, North America faces a shortage of clinical medical physicists, yet there are inadequate slots in the training process of therapy residencies, all competing for the trainee's attention. This is a vicious cycle where staff are increasingly busy in the short term yet unable to commit resources to train the next generation. Accordingly, there is great variability in the brachytherapy training received. U.S. radiation oncologists have residency training stipulations through the Accreditation Council for Graduate Medical Education (ACGME) to actively participate in 15 intracavitary and 12 interstitial cases over four years. This is often accomplished through the residents visiting centers with busy and broad brachytherapy programs. Medical physics residencies accredited by Commission on Accreditation of Medical Physics Education Programs (CAMPEP) last just two years with maybe three months (500 hours) focused on brachytherapy. Given the technical complexity of brachytherapy, which differs markedly from other residency rotation topics, it is clear that many graduates may not have adequate training through the established training pathway. A certified brachytherapy subspecialty within medical physics has been proposed, but training programs and the job market rarely justify having a single, dedicated medical physicist for brachytherapy within most departments.

4. Physics resources and training in low‐ and middle‐income countries are very limited. Many centers around the world still lack the resources to even provide brachytherapy treatments, let alone having high‐quality physics personnel to fully implement those standards. From your perspective, how can international brachytherapy organizations and guideline committees better support resource‐limited clinics while maintaining patient safety and high‐quality physics practice?
  Several global initiatives have been established to address educational gaps and bolster brachytherapy training. The 300‐in‐10 campaign of the American Brachytherapy Society will train 300 teams of radiation oncologists and medical physicists in 10 years with multi‐month intense fellowships generally in North America. GEC‐ESTRO offers the EuroBrachytherapy fellowship program (EuroBT) to provide structured, hands‐on training lasting several months across several European Centers of Excellence. The IAEA leverages the Cyber Learning Platform for Network Education and Training (CLP4Net), offering free online learning modules to guide transition from legacy 2D imaging to modern 3D imaging and treatment planning. Vested industry partners also facilitate peer‐to‐peer training and international site visits to promote safe and high‐quality brachytherapy. However, even the well‐resourced CAMPEP‐accredited medical physicist residency programs described in the prior section remain structurally limited in their dedicated brachytherapy training hours. Physicists in the workplace try balancing brachytherapy specialization while also covering other techniques and procedures offered within radiation oncology. Global harmonization can only occur at a high level, like minimum practice standards, since there is an extraordinary range of available resources and training.

**FIGURE 4:**
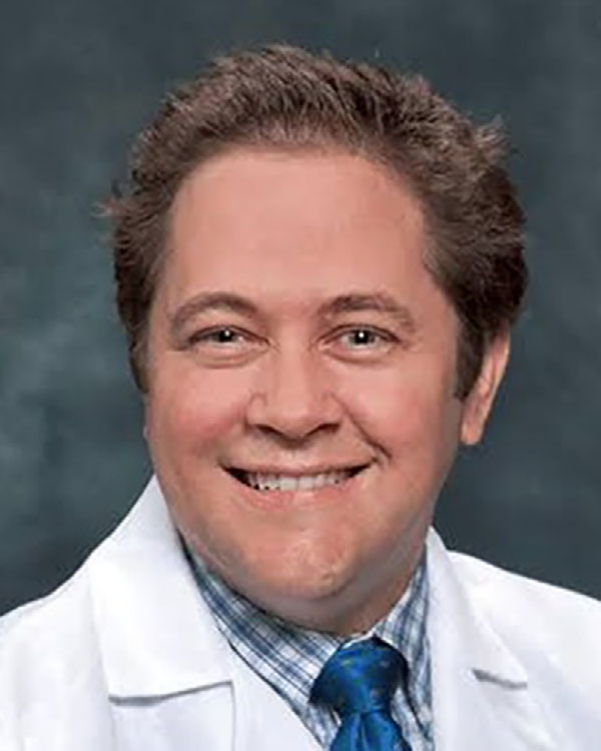
Mark Rivard.

As reflected in the perspectives of four global experts in brachytherapy, Drs. Carlsson Tedgren, Siebert, Rink, and Rivard, exciting technological advancements are emerging in the field of brachytherapy physics, including advances in imaging, in vivo dosimetry, automation, artificial intelligence, MBDCAs, and adaptive treatment approaches. Yet a clear message emerges from these perspectives: technological innovation alone will not overcome the profound global disparities in access to brachytherapy. While these advances can improve efficiency, accuracy, and treatment quality, brachytherapy access continues to depend fundamentally on adequate treatment infrastructure and a skilled workforce.

Bridging the widening gap between increasingly‐ sophisticated technologies and limited access to basic brachytherapy services, particularly in low‐ and middle‐income countries, therefore requires investment not only in innovation, but also in equipment, rigorous physics standards, accessible practical education, and workforce development. As automation becomes increasingly integrated into clinical practice, preserving fundamental physics knowledge, clinical judgment, and meaningful human oversight will remain essential to ensuring safe, effective, and high‐quality patient care.

The future of brachytherapy should therefore be measured not only by technological sophistication, but also by our ability to translate innovation into accessible and sustainable clinical practice. Through international collaboration, resource‐appropriate guidelines, multidisciplinary training, and commitment to equity, brachytherapy can remain an indispensable component of modern radiotherapy and improve patient outcomes for patients worldwide.

## AUTHOR CONTRIBUTIONS

All authors contributed to the conception and development of the manuscript, provided subject‐matter expertise and critical intellectual content, reviewed and revised the manuscript, and approved the final version for publication.

## CONFLICT OF INTEREST STATEMENT

The authors declare no conflicts of interest.
